# *Wt1* haploinsufficiency induces browning of epididymal fat and alleviates metabolic dysfunction in mice on high-fat diet

**DOI:** 10.1007/s00125-021-05621-1

**Published:** 2021-11-30

**Authors:** Karin M. Kirschner, Anna Foryst-Ludwig, Sabrina Gohlke, Chen Li, Roberto E. Flores, Ulrich Kintscher, Michael Schupp, Tim J. Schulz, Holger Scholz

**Affiliations:** 1 Institut für Vegetative Physiologie, Charité-Universitätsmedizin Berlin, corporate member of Freie Universität Berlin, Humboldt-Universität zu Berlin, and Berlin Institute of Health, Berlin, Germany; 2 Institut für Pharmakologie, Charité-Universitätsmedizin Berlin, corporate member of Freie Universität Berlin, Humboldt-Universität zu Berlin, and Berlin Institute of Health, Berlin, Germany; 3 DZHK (German Centre for Cardiovascular Research), partner site Berlin, Berlin, Germany; 4 Department of Adipocyte Development and Nutrition, German Institute of Human Nutrition Potsdam-Rehbrücke, Nuthetal, Germany; 5 Institute of Nutritional Science, University of Potsdam, Potsdam-Rehbrücke, Nuthetal, Germany; 6 German Center for Diabetes Research (DZD), München-Neuherberg, Germany

**Keywords:** Adipocyte, Browning, Glucose tolerance, Hepatic steatosis, High-fat diet, Obesity, Thermogenesis, Transcription factor, UCP1, WT1

## Abstract

**Aims/hypothesis:**

Despite a similar fat storing function, visceral (intra-abdominal) white adipose tissue (WAT) is detrimental, whereas subcutaneous WAT is considered to protect against metabolic disease. Recent findings indicate that thermogenic genes, expressed in brown adipose tissue (BAT), can be induced primarily in subcutaneous WAT. Here, we investigate the hypothesis that the Wilms tumour gene product (WT1), which is expressed in intra-abdominal WAT but not in subcutaneous WAT and BAT, suppresses a thermogenic program in white fat cells.

**Methods:**

Heterozygous *Wt1* knockout mice and their wild-type littermates were examined in terms of thermogenic and adipocyte-selective gene expression. Glucose tolerance and hepatic lipid accumulation in these mice were assessed under normal chow and high-fat diet conditions. Pre-adipocytes isolated from the stromal vascular fraction of BAT were transduced with *Wt1*-expressing retrovirus, induced to differentiate and analysed for the expression of thermogenic and adipocyte-selective genes.

**Results:**

Expression of the thermogenic genes *Cpt1b* and *Tmem26* was enhanced and transcript levels of *Ucp1* were on average more than tenfold higher in epididymal WAT of heterozygous *Wt1* knockout mice compared with wild-type mice. *Wt1* heterozygosity reduced epididymal WAT mass, improved whole-body glucose tolerance and alleviated severe hepatic steatosis upon diet-induced obesity in mice. Retroviral expression of WT1 in brown pre-adipocytes, which lack endogenous WT1, reduced mRNA levels of *Ucp1*, *Ppargc1a*, *Cidea*, *Prdm16* and *Cpt1b* upon in vitro differentiation by 60–90%. WT1 knockdown in epididymal pre-adipocytes significantly lowered *Aldh1a1* and *Zfp423* transcripts, two key suppressors of the thermogenic program. Conversely, *Aldh1a1* and *Zfp423* mRNA levels were increased approximately five- and threefold, respectively, by retroviral expression of WT1 in brown pre-adipocytes.

**Conclusion/interpretation:**

WT1 functions as a white adipocyte determination factor in epididymal WAT by suppressing thermogenic genes. Reducing *Wt1* expression in this and other intra-abdominal fat depots may represent a novel treatment strategy in metabolic disease.

**Graphical abstract:**

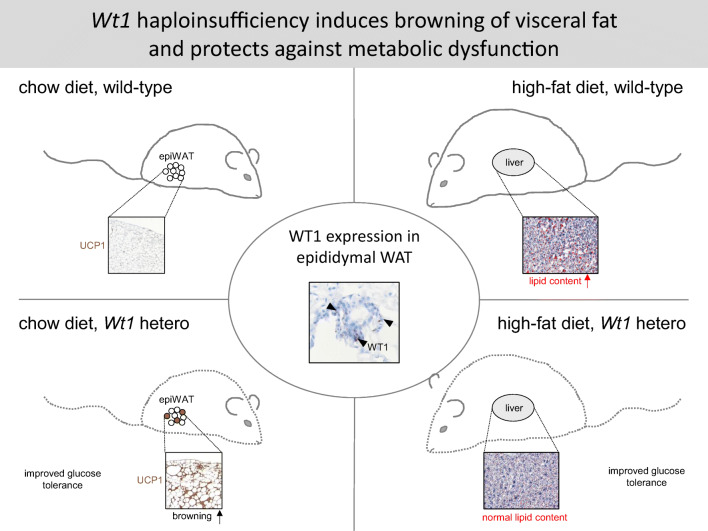

**Supplementary Information:**

The online version of this article (10.1007/s00125-021-05621-1) contains peer-reviewed but unedited supplementary material.

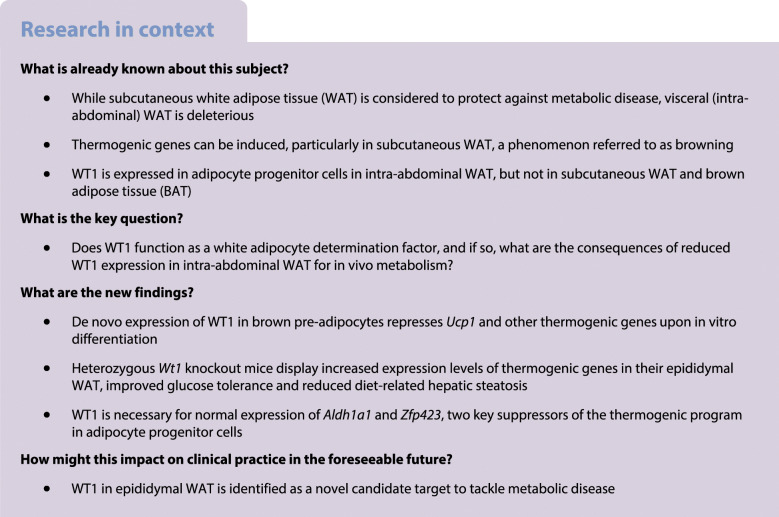



## Introduction

Obesity has emerged as a global health problem that increases the prevalence of many life-shortening disorders including type 2 diabetes, cardiovascular disease, chronic kidney disease and cancer [1, 2]. The adverse effects of obesity are dependent not only on the amount of lipids stored in different fat depots, but also on the site of fat deposition. Lipid accumulation in visceral (intra-abdominal) white adipose tissue (WAT) coincides with increased mortality, even in individuals with normal BMI, whereas subcutaneous obesity is less detrimental [3, 4].

At least two types of brown adipocytes exist besides the energy-storing white fat cells. The classical brown adipose tissue (BAT) fuels energy expenditure by non-shivering thermogenesis [5, 6]. This involves uncoupling protein-1 (UCP1), which dissociates H^+^ fluxes into the mitochondrial matrix from ATP synthesis, thus allowing energy to dissipate as heat [7]. Additionally, a population of brown adipocytes exist in WAT, the so-called beige or brite (brown-in-white) adipocytes, which normally express much lower levels of UCP1 than classical brown fat cells [8–10]. Cold exposure, β-adrenergic agonists and peroxisome proliferator-activated receptor γ (PPARγ) activators can induce thermogenic genes in beige adipocytes, a phenomenon referred to as browning [8, 9, 11, 12]. Adipocyte-specific deletion of the transcriptional co-regulator PR/SET domain 16 (PRDM16) inhibits browning of subcutaneous WAT [13]. Mice without PRDM16 in their white fat cells develop severe insulin resistance and subcutaneous macrophage accumulation on a high-fat diet (HFD), resembling the phenotype of visceral obesity [13]. The appearance of visceral fat-like features in *Prdm16*-deficient subcutaneous adipocytes is also indicated by their reduced thermogenic and enforced proinflammatory gene expression pattern [13].

The transcription factor Wilms tumour gene product (WT1) is expressed de novo in subcutaneous WAT of mice lacking *Prdm16* [13]. WT1 is normally restricted to intra-abdominal and epicardial white fat, which originates from the mesothelium—an epithelial tissue that lines the major body cavities. WT1 was initially identified as a suppressor of renal childhood cancer (Wilms tumour, nephroblastoma). Subsequent gene knockout studies in mice revealed that WT1 is required for embryonic survival and normal development of the genitourinary system and several other tissues [14]. Ubiquitous deletion of *Wt1* in adult mice reduced the intra-abdominal WAT mass, underlining its critical role in the homeostasis of visceral fat [15].

Little is known about the molecular mechanisms that maintain white adipose identity, particularly in the visceral fat. In general, intra-abdominal WAT of mice is largely resistant to browning, whereas beige adipocytes can be readily induced in subcutaneous WAT [11, 16, 17]. The fact that PRDM16 levels in visceral WAT are rather low is unlikely to account for the resistance to browning because transgenic overexpression of *Prdm16* induced brown adipocyte genes in subcutaneous, but not in visceral fat [18]. Instead, additional factors may exist in visceral WAT that limit its susceptibility to browning stimuli.

In this study, we test the hypothesis that WT1, which correlates inversely with PRDM16 levels in differentiating pre-adipocytes [13], determines a white adipose cell fate in epididymal WAT by suppressing thermogenic genes.

## Methods

### Animal care and protocol

All animal experiments were performed in accordance with the Institute for Laboratory Animal Research Guide for Care and Use of Laboratory Animals and approved by the local authorities (permit no. G003/16 and T0308/12 issued by the Landesamt für Gesundheit und Soziales, [LAGeSo], Berlin, Germany). Pathogen-free C57BL/6 J mice with a heterozygous *Wt1* knockout gene (B6;129S4-*Wt1*^*tm1Jae*^/J, The Jackson Laboratory, Bar Harbor, ME, USA) were bred in the local animal house (25°C, 12 h light/dark cycle). Animals were assigned to the specific treatment protocols (see ESM Methods) without randomisation. Blinding of the experiments was not carried out.

### Cell culture and retroviral transductions

Mycoplasma-negative immortalised pre-adipocytes from murine BAT were kindly provided by Y-H Tseng (Joslin Diabetes Center, Harvard Medical School Affiliate, Boston, MA, USA) [19]. Routine maintenance, retroviral transduction and differentiation of the cells are described in ESM Methods [20].

### Isolation and differentiation of precursor cells from murine WAT and BAT

Isolation and maintenance of precursor cells from murine WAT and BAT are reported in ESM Methods [21].

### Silencing of *Wt1* in stromal vascular cells isolated from epididymal WAT

Established protocols were adapted for silencing of *Wt1* in stromal vascular fraction (SVF) cells isolated from the epididymal WAT [22]. See ESM Methods.

### RNA isolation and reverse transcription quantitative PCR

Isolation of total RNA and reverse transcription quantitative PCR (RT-qPCR) from mouse liver and fat, SVF cells and immortalised brown preadipocytes were performed as reported previously [22]. See ESM Methods, ESM Fig. 1. The PCR primer sequences are shown in ESM Table 1.

### SDS-PAGE

Adipose tissues of wild-type and heterozygous *Wt1* knockout mice were lysed in RIPA buffer, and SDS-PAGE was carried-out as described in ESM Methods [23].

### Immunohistochemistry

Immunostaining of formalin-fixed immortalised brown preadipocytes and paraffin-embedded tissue sections (1.5 μm thick) from epididymal WAT and BAT was performed as described elsewhere [24]. See ESM Methods.

### RNAscope

RNAscope (Advanced Cell Diagnostics, Newark, CA, USA) was performed on 1.5 μm thick formalin-fixed, paraffin-embedded tissue sections from epididymal WAT of wild-type mice with *Wt1* probe (#432711) using the RNAscope 2.5 HD Detection Reagent – BROWN Kit (Document No. 322310-USM) according to the manufacturer’s instructions. Probes for dapB (#310043) and Ppib (#313911) were used as negative and positive controls, respectively.

### Determination of the mean adipocyte area

Mean adipocyte area was determined in epididymal WAT of wild-type and heterozygous *Wt1* knockout mice under either chow diet or HFD conditions using Adiposoft within the Fiji software [25]. See ESM Methods.

### H&E and Oil Red O staining

H&E staining was performed on paraffin-embedded adipose tissue following routine protocols [26]. Oil Red O was used for lipid staining [27].

### Quantification of fatty acids in tissues and sera

Triacylglycerols were extracted from homogenised liver tissue from wild-type and heterozygous *Wt1* knockout mice and measured with the colorimetric Triglycerides FS reagent kit (DyaSys Diagnostic Systems, Waterbury, CT, USA). NEFA were determined in sera using the NEFA-HR(2) colorimetric assay kit (Fujifilm, Tokyo, Japan).

### Measurement of glycogen content in the liver

Frozen liver tissue was homogenised with a pestle in liquid nitrogen, dissolved in distilled H_2_O and boiled for 10 min. Glycogen was determined in the supernatant using the Glycogen Colorimetric/Fluorometric Assay kit (BioVision, Milpitas, CA, USA).

### Quantification of serum insulin concentration

Serum insulin was determined using the colorimetric rat/mouse insulin 96-well ELISA kit according to the supplied protocol (Millipore, Burlington, MA, USA).

### Statistics

All data obtained are included in the analyses. Values are presented as means ± SEM. GraphPad PRISM software (Version 9.01) was used for calculation of statistics (GraphPad Software, San Diego, CA, USA). ANOVA with Tukey’s post hoc test and two-tailed Student’s *t* test were performed as indicated. If not otherwise indicated, unpaired Student’s *t* test was used. A *p* value less than 0.05 was considered statistically significant.

## Results

### WT1 suppresses thermogenic genes in differentiating brown precursor cells

**Fig. 1:**
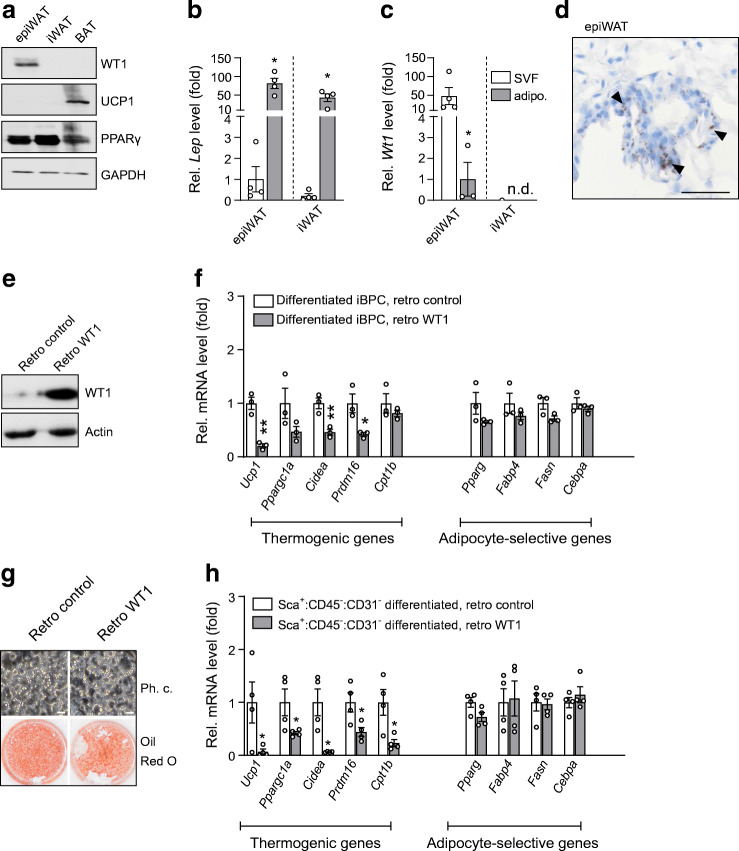
WT1 suppresses thermogenic genes in differentiating brown precursor cells. (**a**) Representative immunoblot of different fat depots from adult male C57BL/6 J mice: epididymal WAT (epiWAT), inguinal WAT (iWAT), interscapular BAT. (**b**, **c**) Relative (Rel.) leptin (*Lep*) (**b**) and *Wt1* (**c**) mRNA levels measured by RT-qPCR in the isolated SVF and the adipocyte (adipo.) fraction of epiWAT and iWAT of adult mice. Leptin mRNA in adipocytes is presented as fold difference vs transcript levels in SVF cells of epiWAT (**b**); *Wt1* transcripts in SVF cells of epiWAT are shown as fold increase vs mRNA levels in adipocytes (**c**). Bars represent means ± SEM, *n* = 4. *p < 0.05 vs SVF, Student’s *t* test; n.d., not detectable. (**d**) *Wt1* mRNA in stromal vascular cells (arrowheads) in murine epiWAT detected by RNAscope. Scale bar, 50 μm. (**e**) Representative immunoblot of immortalised brown pre-adipocytes (iBPC) with (retro WT1) and without (retro control) retroviral expression of WT1. (**f**) Relative mRNA levels in differentiated immortalised brown pre-adipocytes with (black bars) and without (white bars) retroviral overexpression of *Wt1*. Bars represent means ± SEM, *n* = 3. *p < 0.05, **p < 0.01 vs retro control, Student’s *t* test*.* (**g**) Phase contrast (Ph. c.) microscopy and Oil Red O lipid staining of immortalised brown pre-adipocytes. After transduction with either *Wt1*-expressing retrovirus or empty vector retrovirus, the cells were induced to differentiate for 5 days. Scale bars, 20 μm. (**h**) Relative mRNA levels in primary brown adipocytes. Precursor cells were isolated by FACS from the SVF of interscapular BAT of adult mice. Sca1^+^:CD45^−^:CD31^−^ cells were transduced with *Wt1* or empty vector retrovirus, respectively, and grown to confluence for 3 days. Thereafter, the cells were induced to differentiate for 5 days. Transcript levels were measured in differentiated cells by RT-qPCR and normalised to *Actb* mRNA. Bars represent means ± SEM, *n* = 4. *p < 0.05 vs retro control, Student’s *t* test

WT1 protein was detected in epididymal WAT but neither in inguinal (subcutaneous) WAT nor in interscapular BAT (Fig. 1a) [15, 28]. Within epididymal WAT, *Wt1* was highly expressed in the SVF, but not in the leptin-enriched adipocyte fraction (Fig. 1b–d).

To test the hypothesis that WT1 suppresses the BAT genetic program, an immortalised brown pre-adipocyte cell line was transduced with a *Wt1*-expressing retrovirus and subsequently differentiated (Fig. 1e) [19]. While lipid accumulation looked alike (Fig. 1g), *Wt1*-expressing brown adipocytes had significantly lower mRNA levels of the thermogenic genes *Ucp1*, *Cidea* and *Prdm16* than non-expressing cells (Fig. 1f). Transcript levels of adipocyte-selective genes that play a role in both white and brown adipocyte differentiation, i.e. *Pparg*, *Fabp4*, *Fasn* and *Cebpa*, were also slightly reduced in *Wt1* expressing brown adipocytes, but this effect did not reach statistical significance (Fig. 1f).

To validate these findings in primary cultures, we isolated Sca1^+^:CD45^−^:CD31^−^ cells by FACS from the SVF of interscapular BAT of adult mice. Purified cells were transduced with *Wt1-*expressing retrovirus or empty vector and differentiated for 5 days. *Wt1*-expressing brown adipocytes had significantly lower transcript levels of *Ucp1*, *Ppargc1a*, *Cidea*, *Prdm16* and *Cpt1b* compared with *Wt1*-negative cells, but no significant differences in adipocyte-selective transcripts were detected (Fig. 1h). The fraction of Ki-67 immunopositive cells was not significantly different between *Wt1*-expressing and non-expressing brown pre-adipocytes as well as between SVF cells isolated from epididymal WAT of wild-type and heterozygous *Wt1* knockout mice (ESM Fig. 2).

### Heterozygous *Wt1* knockout mice display signs of browning in their epididymal WAT

**Fig. 2:**
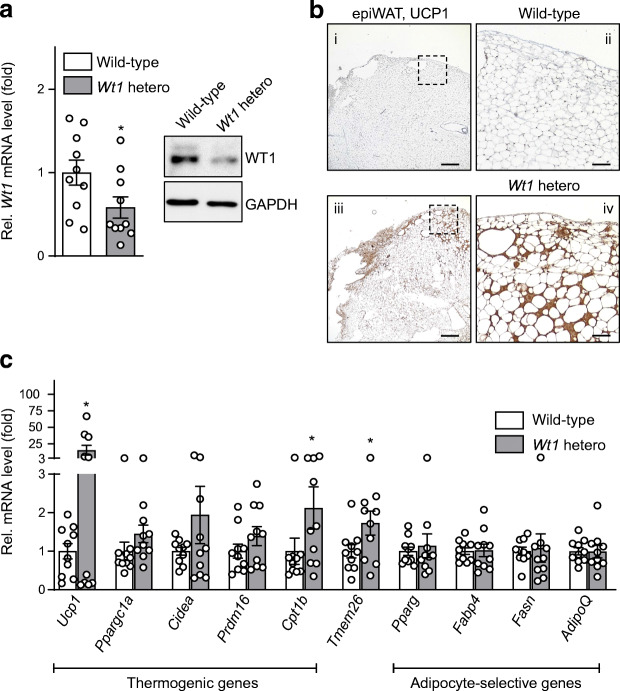
Heterozygous *Wt1* knockout mice display molecular and morphological signs of browning in their epididymal fat. (**a**) Relative (Rel.) *Wt1* mRNA levels (left) and WT1 protein (right) in epididymal WAT (epiWAT) of wild-type and heterozygous *Wt1* knockout mice. Transcript levels were measured by RT-qPCR. Bars indicate means ± SEM, *n* = 10. *p < 0.05 vs wild-type, Student’s *t* test. (**b**) Representative UCP1 immunostaining in epiWAT of a wild-type and a heterozygous *Wt1* knockout mouse (*Wt1* hetero). Scale bars, 400 μm (i, iii) and 100 μm (ii, iv). Panels ii and iv are high power magnifications of the boxed areas in panels i and iii, respectively. (**c**) Relative transcript levels of thermogenic genes, the beige adipocyte gene *Tmem26* and adipocyte-selective genes in epiWAT of wild-type and heterozygous *Wt1* knockout mice. Transcripts were measured by RT-qPCR and normalised to *Actb* mRNA. Data are shown as fold difference between mRNA levels in wild-type and heterozygous *Wt1* knockout mice. Bars indicate means ± SEM, *n* = 10. *p < 0.05 vs wild-type, Student’s *t* test

Heterozygous *Wt1* knockout mice and their wild-type littermates were analysed to explore whether WT1 affects thermogenic gene expression in adipose tissues in vivo. *Wt1* mRNA and protein were lower in epididymal WAT of heterozygous *Wt1* knockout compared with wild-type mice (Fig. 2a). *Wt1* heterozygotes had significantly higher transcript levels of the thermogenic genes *Ucp1* and *Cpt1b*, and the beige adipocyte marker gene *Tmem26* in their epididymal WAT, whereas mRNA levels of adipocyte-selective genes (*Pparg*, *Fabp4*, *Fasn*, *AdipoQ*) were similar (Fig. 2c). Transcript levels of the tested genes were not significantly different in inguinal WAT and interscapular BAT of wild-type and *Wt1* mutant mice (ESM Fig. 3), i.e. in fat depots that do not express *Wt1* (Fig. 1a) [13, 15, 29]. β3-Adrenergic stimulation with CL316,243 (1 μg/g body weight) significantly increased *Ucp1*, *Cidea*, *Cpt1b* and *Adrb3* transcripts in epididymal WAT to a level that was not significantly different (*p* > 0.05, *t* test) between wild-type and heterozygous *Wt1* knockout mice (ESM Fig. 4). In accordance with the increased transcript levels (Fig. 2c), UCP1 immunoreactivity was clearly visible in epididymal WAT of heterozygous *Wt1* knockout mice, but not of wild-type mice (Fig. 2b). Interscapular BAT was used as a positive control for UCP1 immunostaining (ESM Fig. 5a). To corroborate the in vivo findings in cell culture, SVF cells were freshly isolated from epididymal WAT of wild-type and heterozygous *Wt1* knockout mice, expanded and induced to differentiate into adipocytes. All cultures looked similar in terms of cellular lipid accumulation. Notably, adipogenic differentiation was associated with an increase in *Ucp1* mRNA levels that was statistically significant in cells obtained from heterozygous *Wt1* knockout mice (ESM Fig. 5b).

### Heterozygous *Wt1* knockout mice receiving HFD have a lower epididymal WAT to body weight ratio

**Fig. 3:**
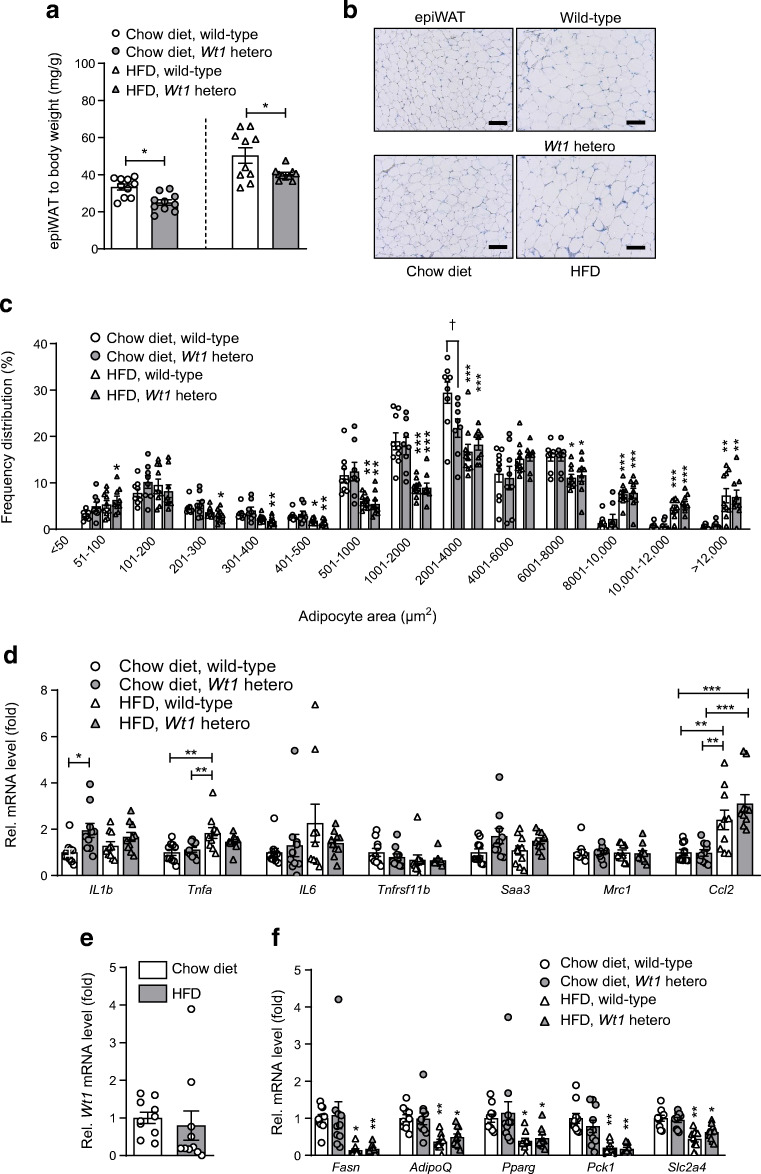
Heterozygous *Wt1* knockout mice fed with HFD have lower epididymal WAT to body weight ratios. Wild-type and heterozygous *Wt1* knockout mice (*n* = 40 total) were kept on either chow diet (10% of kJ from fat) or HFD (60% of kJ from fat) for 11 weeks. (**a**) Epididymal WAT (epiWAT) weight to body weight ratios. Bars indicate means ± SEM, *n* = 10, each. *p < 0.05 between wild-type and *Wt1* mutant animals (Student’s *t* test). Note that, for better data visualisation, statistical significance between mice receiving chow diet and HFD is not indicated. (**b**) Representative H&E staining of epiWAT of a wild-type and heterozygous *Wt1* knockout mouse fed with either chow or HFD. Scale bars, 100 μm. (**c**) Frequency distribution of adipocyte areas in epididymal WAT of wild-type and heterozygous *Wt1* knockout mice receiving either chow diet or HFD. Measurements were performed with tissue sections from *n* = 40 animals analysing more than 2500 cells per group. Statistical differences between mice of identical genotype receiving HFD vs chow diet are indicated by asterisks (*p < 0.05, **p < 0.01, ***p < 0.001, ANOVA with Tukey post hoc test). ^†^*p* < 0.05, statistical differences between wild-type and heterozygous *Wt1* knockout mice (ANOVA with Tukey post hoc test). Note that the adipocyte areas were not significantly different between wild-type and heterozygous *Wt1* knockout mice except for the size range 2001–4000 μm^2^. In this particular range, *Wt1* knockout mice fed with chow diet had significantly smaller adipocytes in their epididymal WAT than wild-type mice. (**d**) Relative mRNA levels of inflammation-related genes in epididymal WAT of normal and *Wt1* mutant mice. Bars represent means ± SEM, *n* = 10 in each group. *p < 0.05, **p < 0.01 and ***p < 0.001 as shown, ANOVA with Tukey post hoc test. (**e**) Relative *Wt1* mRNA levels in epididymal WAT of wild-type mice receiving either chow diet or HFD. Transcripts were measured by RT-qPCR and normalised to *Actb* mRNA. Bars indicate means ± SEM, *n* = 10. (**f**) Relative transcript levels of genes involved in fatty acid and glucose homeostasis in epididymal WAT of wild-type and heterozygous *Wt1* knockout mice receiving either normal diet or HFD. Bars show means ± SEM, *n* = 10 in each group. *p < 0.05 and **p < 0.01 between mice of the same genotype receiving HFD vs chow diet, ANOVA with Tukey post hoc test

To test whether wild-type and heterozygous *Wt1* knockout mice respond differently to metabolic stress conditions, they received either HFD or chow diet for 11 weeks. Minor differences in daily food intake, body weight, relative lean body mass, serum concentrations of insulin and NEFA and locomotor activity were observed between wild-type and heterozygous *Wt1* knockout mice (ESM Fig. 6). Mice with a single intact *Wt1* allele had a significantly lower epididymal WAT to body weight ratio (Fig. 3a). As expected, mean adipocyte areas were larger in epididymal WAT of mice receiving HFD vs chow diet (Fig. 3b, c). However, significant differences in adipocyte areas were generally not observed between wild-type and heterozygous *Wt1* knockout mice except for the range from 2001 to 4000 μm^2^. In this size range, the adipocytes of chow-fed heterozygous *Wt1* knockout mice were significantly smaller than those of wild-type mice (Fig. 3c). *Il1b* transcripts were approximately twofold higher in epididymal WAT of heterozygous *Wt1* knockout mice than wild-type mice on chow diet (Fig. 3d). *Tnfa* mRNA levels in epididymal WAT increased upon HFD feeding, and this effect was significant (*p* < 0.01) in wild-type but not in *Wt1* mutant mice (Fig. 3d). *Wt1* transcripts were slightly (*p* > 0.05) higher with a broader value distribution in HFD-fed animals (Fig. 3e). Transcript levels of genes involved in fatty acid and glucose metabolism, i.e. *Fasn*, *AdipoQ*, *Pparg*, *Pck1* and *Slc2a4*, were significantly lower in epididymal WAT of HFD- vs chow-fed mice (Fig. 3f). Heterozygous *Wt1* knockout mice on HFD had higher *Ucp1* mRNA levels in their epididymal WAT than wild-type mice (ESM Fig. 7).

### Heterozygous *Wt1* knockout mice show improved whole-body glucose tolerance and reduced hepatic steatosis

**Fig. 4:**
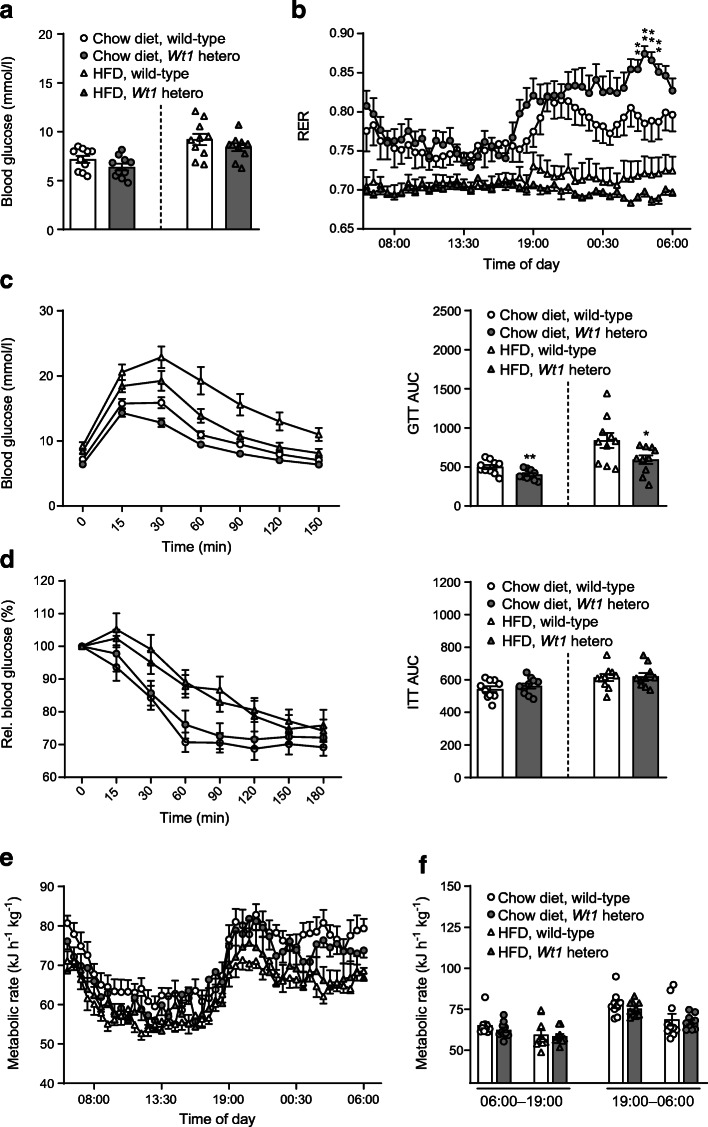
Heterozygous *Wt1* knockout mice show improved whole-body glucose tolerance. Fasting blood glucose levels (**a**) and RER (**b**) in wild-type and *Wt1* mutant mice kept on either chow diet or HFD for 11 weeks. Asterisks indicate statistical differences between wild-type and heterozygous *Wt1* knockout mice fed with chow diet. **p < 0.01, ANOVA with Tukey post hoc test, *n* = 10. Glucose (**c**) and insulin (**d**) tolerance test. Values are means ± SEM, *p < 0.05, **p < 0.01 vs wild-type, Student’s *t* test, *n* = 10 in each group. Diurnal (**e**) and phasic (**f**) metabolic rates of wild-type and *Wt1* mutant mice on HFD and chow diet

We reasoned that enhanced *Ucp1* expression in epididymal WAT might protect heterozygous *Wt1* knockout mice against HFD-induced metabolic dysfunction. Animals receiving HFD had indeed higher blood glucose concentrations than mice on chow diet, but no significant differences were observed between wild-type and heterozygous *Wt1* knockout mice (Fig. 4a). The respiratory exchange ratio (RER, $\dot{V}\mathrm{C}{\mathrm{O}}_2$/$\dot{V}{\mathrm{O}}_2$) was elevated between 03:00 h and 05:30 h in chow-fed *Wt1* heterozygotes suggesting that carbohydrates were their predominant fuel source during the final stages of the dark phase (Fig. 4b). *Wt1* mutant mice showed a significantly improved whole-body glucose tolerance (Fig. 4c). Serum insulin concentrations (ESM Fig. 6e) and insulin sensitivity (Fig. 4d) were not significantly different between normal and *Wt1* mutant mice. Metabolic rates followed a circadian rhythm with higher values during the dark period (Fig. 4e, f).

**Fig. 5:**
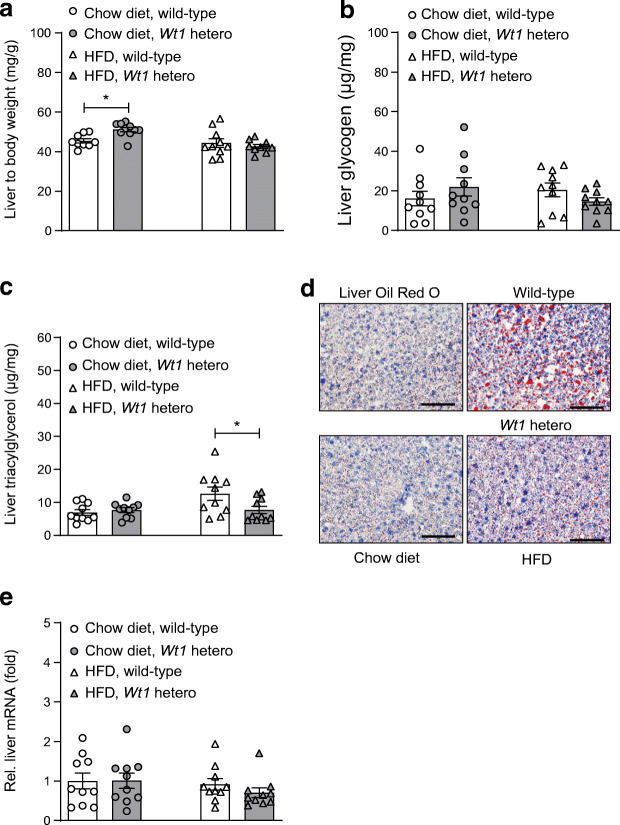
Heterozygous *Wt1* knockout mice on HFD show reduced hepatic steatosis. Liver to body weight ratio (**a**), hepatic glycogen (**b**) and triacylglycerol content per milligram liver tissue (**c**) of wild-type and heterozygous *Wt1* knockout mice fed with either chow diet or HFD. *p < 0.05, ANOVA with Tukey post hoc test. Note that for better data visualisation, statistical significance between mice receiving chow diet and HFD is not indicated. (**d**) Representative Oil Red O lipid staining of liver sections from wild-type and heterozygous *Wt1* knockout mice receiving either chow diet or HFD. Scale bars, 100 μm. (**e**) *Wt1* transcript levels measured by RT-qPCR and normalised to *Actb* mRNA in the livers of wild-type mice receiving either chow diet or HFD. Bars represent means ± SEM, *n* = 10. *p* > 0.05, ANOVA with Tukey post hoc test

Heterozygous *Wt1* knockout mice on chow diet, but not on HFD, had significantly higher liver to body weight ratios than their wild-type littermates (Fig. 5a). The amount of glycogen stored per gram liver tissue was not significantly different between the groups (Fig. 5b). Hepatic triacylglycerol content was similar in wild-type and *Wt1* mutant mice on chow diet (Fig. 5c). HFD feeding increased triacylglycerol levels nearly twofold in the livers of wild-type but not of *Wt1* mutant mice (Fig. 5c). Accordingly, wild-type mice showed severe hepatic steatosis with clearly milder changes in the *Wt1* heterozygotes (Fig. 5d). HFD feeding did not significantly alter *Wt1* mRNA levels in the liver of wild-type and *Wt1* mutant mice (Fig. 5e).

### WT1 increases *Aldh1a1* and *Zfp423* expression in adipogenic precursor cells

**Fig. 6:**
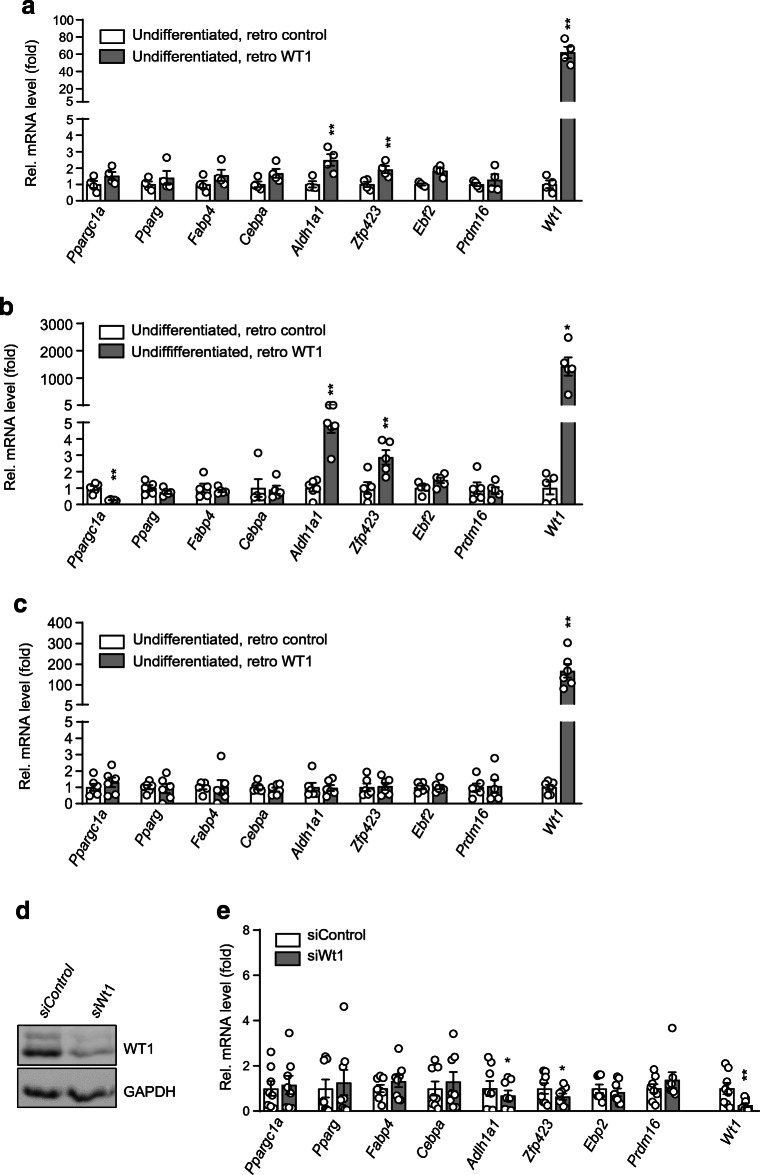
*Wt1* increases *Aldh1a1* and *Zfp423* expression in adipogenic precursor cells. (**a**) Relative mRNA levels of genes involved in adipose cell fate determination in SVF cells isolated from interscapular BAT of adult mice. Cells were transduced with either *Wt1*-expressing retrovirus (retro WT1) or empty vector control (retro control). (**b**) Transcript levels in immortalised brown pre-adipocytes with and without retroviral expression of WT1. (**c**) Relative mRNA levels in *Wt1*-expressing and non-expressing SVF cells isolated from inguinal WAT of adult mice. WT1 immunoblot (**d**) and relative transcript levels (**e**) of undifferentiated SVF cells prepared from epididymal WAT of adult mice. Primary cells at approximately 50% confluence were incubated with either non-targeting control siRNA or *Wt1* siRNA for 48 h. Transcript levels were measured by RT-qPCR and normalised to *Actb*. In each figure part, mRNA levels are shown as fold difference between cells transfected with *Wt1* siRNA (siWt1) and non-targeting siRNA (siControl). Bars represent means ± SEM, *n* = 4 (**a**), *n* = 5 (**b**), *n* = 6 (**c**) and *n* = 8 (**e**). *p < 0.05, **p < 0.01, Student’s paired *t* test. Note that all data shown were obtained with undifferentiated cells

Finally, we sought to identify potential downstream pathways, along which WT1 may repress a brown adipocyte differentiation program. Notably, transcript levels of *Aldh1a1* and *Zfp423*, two key determinants of white adipose identity [30–32], were significantly increased upon retroviral expression of *Wt1* in undifferentiated SVF cells from interscapular BAT and in immortalised brown pre-adipocytes (Fig. 6a, b). *Wt1* expressing pre-adipocytes also had reduced mRNA levels of *Ppargc1a* (Fig. 6b), which interacts with nuclear receptor PPARγ in the regulation of BAT mitochondrial biogenesis [33]. Retroviral delivery of WT1 did not affect mRNA levels in SVF cells isolated from inguinal WAT (Fig. 6c). Silencing of *Wt1* significantly reduced *Aldh1a1* and *Zfp423* transcripts in SVF cells from epididymal WAT (Fig. 6d, e).

## Discussion

Visceral adipose tissue of mice is normally less susceptible to browning than subcutaneous WAT [34, 35]. Our current data suggest that WT1, which is expressed in epididymal but not in subcutaneous WAT (Fig. 1a, [28]), is a direct or indirect repressor of WAT browning. Inhibition of WAT browning by WT1 in vivo is indicated by the upregulation of thermogenic genes in epididymal WAT of heterozygous *Wt1* knockout mice (Fig. 2). Remarkably, β3-adrenergic stimulation with CL316,243 eliminates differences in thermogenic gene expression between wild-type and *Wt1* mutant mice (ESM Fig. 4). This suggests that the repressor activity of WT1 on WAT browning can be antagonised by the effect of a potent β3-adrenergic stimulus. Hence, WT1 does not limit the overall browning capacity of epididymal WAT, but may rather contribute to the maintenance of WAT identity under unchallenging conditions, i.e. in the absence of a strong browning inducer.

Silencing of endogenous *Wt1* in epididymal SVF cells reduces *Aldh1a1* and *Zfp423* transcripts, whereas retroviral expression of *Wt1* increases these mRNAs in brown precursor cells (Fig. 6). These findings suggest that WT1 stimulates *Aldh1a1* and *Zfp423* expression in epididymal pre-adipocytes either directly or through indirect mechanisms. Deletion of either *Aldh1a1* or *Zfp423* has been reported to induce thermogenic genes in visceral fat depots [30, 32, 36]. Retinal dehydrogenase 1 (ALDH1A1) is the rate-limiting enzyme in the conversion of retinaldehyde (Rald) to retinoic acid. *Aldh1a1* deficiency increases Rald concentrations, particularly in tissues with high endogenous *Aldh1a1* expression, such as WAT [37, 38]. By activating retinoic acid receptor (RAR)-dependent mechanisms, Rald functions as a positive transcriptional regulator of the classical BAT program in white fat cells [30]. Zinc finger protein 423 (Zfp423) functions as a transcription factor that maintains white adipocyte identity by suppressing thermogenic genes [31, 32]. It acts, at least partially, by inhibiting the activity of Ebf2 and thereby represses *Prdm16* [32]. Remarkably, *Zfp423* was among the top 1000 genes (*p* value 4.7 × 10^−23^) bound by WT1 in a genome-wide ChIP-sequencing analysis of mouse embryonic kidneys [39].

Importantly, retroviral delivery of WT1 in brown precursor cells does not inhibit adipogenic differentiation in general nor does WT1 significantly downregulate adipocyte-selective genes that are involved in white and brown adipocyte differentiation (Fig. 1). Instead, ectopic WT1 predominantly represses thermogenic genes in differentiating brown precursor cells (Fig. 1e, g). One can therefore assume that the brown adipose signature in epididymal WAT of heterozygous *Wt1* knockout mice is genuine and not merely due to subtle developmental defects. Furthermore, these findings qualify WT1 as a repressor of brown adipocyte identity.

What are possible functional consequences arising from a BAT-like gene expression pattern in the epididymal fat of heterozygous *Wt1* knockout mice? Strikingly, mice with a heterozygous *Wt1* gene show improved whole-body glucose tolerance and alleviated diet-induced fatty liver disease (Figs 4, 5). The former observation is in keeping with the higher RER of heterozygous *Wt1* mutant mice indicating that carbohydrates are their predominant fuel source on HFD, at least during the last phase of diurnal physical activity (Fig. 4b). Activation of classical BAT and browning of WAT are associated with a favourable metabolic status including improved insulin sensitivity and reduced fatty liver disease [5, 40–42]. Accordingly, inhibition of beige adipocyte function by conditional deletion of *Prdm16* caused severe insulin resistance and hepatic steatosis in mice receiving HFD [13]. Thus, we assume that thermogenic genes expressed in epididymal WAT of heterozygous *Wt1* knockout mice might contribute to their improved glucose tolerance and largely preserved liver morphology upon HFD feeding. However, a causative relationship between epididymal WAT browning and metabolic function in *Wt1* mutant mice cannot be derived from our data. Our findings also do not allow us to exclude that preserved metabolic function is secondary to *Wt1* heterozygosity in the liver or other tissues. More specific approaches using conditional *Wt1* knockout mice are necessary to resolve this important issue.

HFD feeding does not significantly increase *Wt1* mRNA levels in murine epididymal WAT (Fig. 3e). This observation is in agreement with transcriptome analyses in humans reporting similar *Wt1* mRNA levels in the visceral fat of lean and obese individuals [43]. Surprisingly, body weight gain and metabolic rates are not significantly different between wild-type and *Wt1* mutant mice (Fig. 4e, f, ESM Fig. 6b, c). This might seem puzzling at first sight as browning of subcutaneous WAT is normally associated with increased energy expenditure and reduced obesity in mice [44–46]. However, classical inducers of WAT browning, such as exposure to low temperature, PPARγ agonists or β-adrenergic stimulation usually increase thermogenic genes in subcutaneous WAT to much higher levels than those measured in epididymal WAT of heterozygous *Wt1* knockout mice [8, 11, 13]. One can therefore speculate whether the induction of thermogenic genes in epididymal WAT of *Wt1* mutant mice is not strong enough for eliciting a significant increase in whole-body energy expenditure in the conditions tested.

In summary, our findings demonstrate that *Wt1* haploinsufficiency activates a brown adipocyte genetic program in epididymal WAT of mice. We suggest that browning of epididymal WAT depots contributes to reduced diet-related hepatic steatosis and increased whole-body glucose tolerance in mice with a single intact *Wt1* allele. Thus, our results identify WT1 as a repressor of brown adipocyte identity and a potential therapeutic target in metabolic disorders.

## Supplementary information

ESM (PDF 1.37 MB)

## Data Availability

Data are available on request from the authors.

## Abbreviations

BAT: Brown adipose tissue
HFD: High-fat diet
PPARγ: Peroxisome proliferator-activated receptor γ
PRDM16: PR/SET domain 16
RER: Respiratory exchange ratio
RT-qPCR: Reverse transcription quantitative PCR
SVF: Stromal vascular fraction
UCP1: Uncoupling protein-1
WAT: White adipose tissue
WT1: Wilms tumour gene product

## References

[1] Bray GA, Bellanger T (2006). Epidemiology, trends, and morbidities of obesity and the metabolic syndrome. Endocrine.

[2] Mensah GA, Mokdad AH, Ford E (2004). Obesity, metabolic syndrome, and type 2 diabetes: emerging epidemics and their cardiovascular implications. Cardiol Clin.

[3] Pischon T, Boeing H, Hoffmann K (2008). General and abdominal adiposity and risk of death in Europe. N Engl J Med.

[4] Manolopoulos KN, Karpe F, Frayn KN (2010). Gluteofemoral body fat as a determinant of metabolic health. Int J Obes.

[5] Cypess AM, Kahn CR (2010). The role and importance of brown adipose tissue in energy homeostasis. Curr Opin Pediatr.

[6] Cannon B, Nedergaard J (2004). Brown adipose tissue: function and physiological significance. Physiol Rev.

[7] Nedergaard J, Golozoubova V, Matthias A, Asadi A, Jacobsson A, Cannon B (2001). UCP1: the only protein able to mediate adaptive non-shivering thermogenesis and metabolic inefficiency. Biochim Biophys Acta.

[8] Petrovic N, Walden TB, Shabalina IG, Timmons JA, Cannon B, Nedergaard J (2010). Chronic peroxisome proliferator-activated receptor gamma (PPARgamma) activation of epididymally derived white adipocyte cultures reveals a population of thermogenically competent, UCP1-containing adipocytes molecularly distinct from classic brown adipocytes. J Biol Chem.

[9] Wu J, Bostrom P, Sparks LM (2012). Beige adipocytes are a distinct type of thermogenic fat cell in mouse and human. Cell.

[10] Ishibashi J, Seale P (2010). Medicine. Beige can be slimming. Science.

[11] Ohno H, Shinoda K, Spiegelman BM, Kajimura S (2012). PPARgamma agonists induce a white-to-brown fat conversion through stabilization of PRDM16 protein. Cell Metab.

[12] Herz CT, Kiefer FW (2019). Adipose tissue browning in mice and humans. J Endocrinol.

[13] Cohen P, Levy JD, Zhang Y (2014). Ablation of PRDM16 and beige adipose causes metabolic dysfunction and a subcutaneous to visceral fat switch. Cell.

[14] Kreidberg JA, Sariola H, Loring JM (1993). WT-1 is required for early kidney development. Cell.

[15] Chau YY, Brownstein D, Mjoseng H (2011). Acute multiple organ failure in adult mice deleted for the developmental regulator Wt1. PLoS Genet.

[16] Vitali A, Murano I, Zingaretti MC, Frontini A, Ricquier D, Cinti S (2012). The adipose organ of obesity-prone C57BL/6J mice is composed of mixed white and brown adipocytes. J Lipid Res.

[17] Zuriaga MA, Fuster JJ, Gokce N, Walsh K (2017). Humans and mice display opposing patterns of "Browning" gene expression in visceral and subcutaneous white adipose tissue depots. Front Cardiovasc Med.

[18] Seale P, Conroe HM, Estall J (2011). Prdm16 determines the thermogenic program of subcutaneous white adipose tissue in mice. J Clin Invest.

[19] Tseng YH, Butte AJ, Kokkotou E (2005). Prediction of preadipocyte differentiation by gene expression reveals role of insulin receptor substrates and necdin. Nat Cell Biol.

[20] Gohlke S, Zagoriy V, Cuadros Inostroza A (2019). Identification of functional lipid metabolism biomarkers of brown adipose tissue aging. Mol Metab.

[21] Steinbring J, Graja A, Jank AM, Schulz TJ (2017). Flow cytometric isolation and differentiation of Adipogenic progenitor cells into Brown and Brite/beige adipocytes. Methods Mol Biol.

[22] Kirschner KM, Braun JF, Jacobi CL, Rudigier LJ, Persson AB, Scholz H (2014). Amine oxidase copper-containing 1 (AOC1) is a downstream target gene of the Wilms tumor protein, WT1, during kidney development. J Biol Chem.

[23] Muller M, Persson AB, Krueger K, Kirschner KM, Scholz H (2017). The Wilms tumor protein WT1 stimulates transcription of the gene encoding insulin-like growth factor binding protein 5 (IGFBP5). Gene.

[24] Kirschner KM, Sciesielski LK, Krueger K, Scholz H (2017). Wilms tumor protein-dependent transcription of VEGF receptor 2 and hypoxia regulate expression of the testis-promoting gene Sox9 in murine embryonic gonads. J Biol Chem.

[25] Galarraga M, Campión J, Muñoz-Barrutia A (2012). Adiposoft: automated software for the analysis of white adipose tissue cellularity in histological sections. J Lipid Res.

[26] Fischer AH, Jacobson KA, Rose J, Zeller R (2008). Hematoxylin and eosin staining of tissue and cell sections. CSH Protoc.

[27] Mehlem A, Hagberg CE, Muhl L, Eriksson U, Falkevall A (2013). Imaging of neutral lipids by oil red O for analyzing the metabolic status in health and disease. Nat Protoc.

[28] Chau YY, Bandiera R, Serrels A (2014). Visceral and subcutaneous fat have different origins and evidence supports a mesothelial source. Nat Cell Biol.

[29] Lee KY, Luong Q, Sharma R, Dreyfuss JM, Ussar S, Kahn CR (2019). Developmental and functional heterogeneity of white adipocytes within a single fat depot. EMBO J.

[30] Kiefer FW, Vernochet C, O'Brien P (2012). Retinaldehyde dehydrogenase 1 regulates a thermogenic program in white adipose tissue. Nat Med.

[31] Gupta RK, Arany Z, Seale P (2010). Transcriptional control of preadipocyte determination by Zfp423. Nature.

[32] Shao M, Ishibashi J, Kusminski CM (2016). Zfp423 maintains white adipocyte identity through suppression of the beige cell thermogenic gene program. Cell Metab.

[33] Puigserver P, Wu Z, Park CW, Graves R, Wright M, Spiegelman BM (1998). A cold-inducible coactivator of nuclear receptors linked to adaptive thermogenesis. Cell.

[34] Guerra C, Koza RA, Yamashita H, Walsh K, Kozak LP (1998). Emergence of brown adipocytes in white fat in mice is under genetic control. Effects on body weight and adiposity. J Clin Invest.

[35] Walden TB, Hansen IR, Timmons JA, Cannon B, Nedergaard J (2012). Recruited vs. nonrecruited molecular signatures of brown, "brite," and white adipose tissues. Am J Physiol Endocrinol Metab.

[36] Hepler C, Shao M, Xia JY et al (2017) Directing visceral white adipocyte precursors to a thermogenic adipocyte fate improves insulin sensitivity in obese mice. Elife 6. 10.7554/eLife.27669 10.7554/eLife.27669 PMC5552276 28722653

[37] Ziouzenkova O, Orasanu G, Sharlach M (2007). Retinaldehyde represses adipogenesis and diet-induced obesity. Nat Med.

[38] Molotkov A, Duester G (2003). Genetic evidence that retinaldehyde dehydrogenase Raldh1 (Aldh1a1) functions downstream of alcohol dehydrogenase Adh1 in metabolism of retinol to retinoic acid. J Biol Chem.

[39] Motamedi FJ, Badro DA, Clarkson M (2014). WT1 controls antagonistic FGF and BMP-pSMAD pathways in early renal progenitors. Nat Commun.

[40] Betz MJ, Enerback S (2018). Targeting thermogenesis in brown fat and muscle to treat obesity and metabolic disease. Nat Rev Endocrinol.

[41] Choi SS, Kim ES, Jung JE (2016). PPARgamma antagonist Gleevec improves insulin sensitivity and promotes the Browning of white adipose tissue. Diabetes.

[42] Kroon T, Harms M, Maurer S (2020). PPARγ and PPARα synergize to induce robust browning of white fat in vivo. Mol Metab.

[43] Yang L, Wang X, Guo H, Zhang W, Wang W, Ma H (2019). Whole transcriptome analysis of obese adipose tissue suggests u001kfc.1 as a potential regulator to glucose homeostasis. Front Genet.

[44] Bartelt A, Heeren J (2014). Adipose tissue browning and metabolic health. Nat Rev Endocrinol.

[45] Harms M, Seale P (2013). Brown and beige fat: development, function and therapeutic potential. Nat Med.

[46] Kajimura S, Spiegelman BM, Seale P (2015). Brown and Beige fat: physiological roles beyond heat generation. Cell Metab.

