# Supplementary material for: Wt1 haploinsufficiency induces browning of epididymal fat and alleviates metabolic dysfunction in mice on high-fat diet
Source: Diabetologia. 2021 Nov 30;65(3):528–40. doi: 10.1007/s00125-021-05621-1 (PMC8803700; doi:10.1007/s00125-021-05621-1)
Supplement: Supplementary file 1 — (PDF 1.37 MB) [file 125_2021_5621_MOESM1_ESM.pdf]

Wt1 haploinsufficiency induces browning of epididymal fat and alleviates metabolic dysfunction in mice on high-fat diet

Karin M. Kirschner, Anna Foryst-Ludwig, Sabrina Gohlke, Chen Li, Roberto E. Flores, Ulrich Kintscher, Michael Schupp, Tim J. Schulz and Holger Scholz

Electronic Supplementary Material

ESM Methods

Animal care and protocol

Male mice (C57BL/6J) between 8-10 months old at the beginning of the experimental protocol were used throughout. Wild-type mice and littermates with a heterozygous *Wt1* gene were fed either chow (10% of kJ from fat, 16.11 kJ/g chow, D12450B, Research Diets, Inc., New Brunswick, NJ, USA) or a high fat diet (HFD, 60% of kJ from fat, 21.92 kJ/g chow, D12492, Research Diets, Inc., New Brunswick, NJ, USA) for 11 weeks. All animals (n=10 per group) had free access to food and fresh drinking water. Body weight and food intake were determined every 3 days throughout the experiment. The body composition was analysed at the beginning and the end of the treatment protocol by nuclear magnetic resonance imaging (Bruker's Minispec MQ10, Bruker Corporation, Billerica, MA, USA) as described.(1) After 8 weeks of feeding with either HFD or chow diet, heterozygous *Wt1* knockout mice and their wild-type littermates were analysed for energy expenditure, respiratory exchange ratio (RER) and locomotor activity using a custom-made 4-cage calorimetry system (LabMaster, TSE Systems, Bad Homburg von der Höhe, Germany) as reported in detail elsewhere.(1) Recordings were performed for 24 h with 7 mice per group. After 10 weeks, glucose tolerance testing (GTT) was performed on overnight-fasted animals by intraperitoneal injection of glucose (1 g/kg body weight). Blood was collected from the tail veins for glucose quantification with a glucometer (Precision Xtra; Abbott, Chicago, IN, USA). An insulin tolerance test (ITT) with intraperitoneally injected 0.5 units/kg body weight insulin (Actrapid; Novo Nordisk, Bagsværd, Denmark) was performed after 11 weeks.(1) Areas und the curves (AUCs) were calculated with subtraction of baseline values for each single animal using the GraphPad PRISM software (Version 9.01). Immediately after ITT, the animals were killed under isoflurane anaesthesia and their organs dissected. Some wild-type and heterozygous *Wt1* knockout mice were injected intraperitoneally with the β3-adrenergic agonist CL316,243 (Sigma-Aldrich, St. Louis, MO, USA) on 10 consecutive days. The daily administered dose of CL316,243 was 1 µg/ g body weight corresponding to 10 µl/ g body weight of compound dissolved in PBS.

Cell culture and retroviral transductions

Immortalised preadipocytes from murine BAT were routinely cultivated in Dulbecco's modified Eagle's medium (DMEM) supplemented with 10% fetal bovine serum (Gibco, Thermo Fisher Scientific Inc., Waltham, MA, USA), 20 mM Hepes buffer and 1% penicillin-streptomycin. The confluent cells were induced for 48 h in medium containing 2% fetal bovine serum, 20 nmol/l human recombinant insulin, 0.125 mmol/l indomethacin, 0.5 mmol/l

isobutylmethylxanthine (IBMX), 5 µmol/l dexamethasone and 1 nmol/l T3 as described.(2) The cells were differentiated from day 5 to day 10 in medium (DMEM, 2% fetal bovine serum, 20 mmol/l Hepes buffer, 1% penicillin-streptomycin) with 20 nmol/l insulin and 1 nmol/l T3.(2) If not otherwise indicated, all chemical compounds were obtained from Sigma-Aldrich.

Isolation and differentiation of precursor cells from murine WAT and BAT

The primary cells representing the stromal vascular fraction (SVF) of adipose tissue were grown in Dulbecco's modified Eagle's medium (DMEM) supplemented with 10% fetal bovine serum (growth medium), 20 mmol/l Hepes buffer and 1% penicillin-streptomycin (Gibco). In some experiments, the freshly isolated SVF cells were resuspended in sorting medium (2% fetal bovine serum in HBSS) and purified by flow cytometry with the following antibodies that are widely used for this purpose: 0.5 µg/ml anti-mouse-Sca1 labelled with APC (clone D7; isotype Rat IgG2a, eBioscience), 2.5 µg/ml anti-mouse-CD45 labelled with FITC (clone 30-F11; isotype Rat IgG2b, eBioscience), 1 µg/ml anti-mouse-CD31 labelled with FITC (clone 390; isotype Rat IgG2a, BioLegend, San Diego, CA, USA). $Sca1^{+}/Lin^{-}$ cells were selected and grown in Dulbecco's modified Eagle's medium (DMEM) supplemented with 10% fetal bovine serum (growth medium), 20 mmol/l Hepes buffer and 1% penicillin-streptomycin (Gibco). Retroviral transductions were performed at approximately 40% confluence. Transduced precursor cells were grown to confluence and afterwards induced for 2 days in growth medium supplemented with 20 nmol/l human insulin, 50 µmol/l indomethacin, 0.5 µmol/l dexamethasone, 0.5 mmol/l IBMX and 1 nmol/l T3. Subsequently, differentiation was performed for 5 days in growth medium supplemented with 20 nmol/l human insulin and 1 nmol/l T3.(2)

Silencing of *Wt1* in stromal vascular (SVF) cells isolated from epididymal WAT

Primary SVF cells were grown to approximately 60% confluence. A pool of four different siRNAs (5.26 pmol/cm^2, Dharmacon, Thermo Fisher Scientific, Darmstadt, Germany) targeting the murine *Wt1* gene (ON-TARGETplus, SMARTpool siRNA, L-040686-01-0005, NM_144783) was transfected with 1.04 µl/cm^2 of the DharmaFECT 1 reagent (Dharmacon).(3) Four different non-targeting siRNAs (siGENOME non-targeting siRNA pool 2, Dharmacon) were used as negative control. The cells were harvested 48 hours after siRNA transfection.

RNA isolation and reverse transcription (RT) quantitative PCR

Total RNA was isolated from mouse liver, SVF cells and immortalised brown preadipocytes using the RNAbee reagent (AMSBIO, Abingdon, UK). Mouse adipose tissue was homogenised

with QIAzol Lysis Reagent (Qiagen, Venlo, Netherlands) for isolation of total RNA, which was subsequently purified with the RNeasy Micro Kit (Qiagen). First-strand cDNA synthesis was performed with 1 µg of total RNA using oligo(dT) primers and Superscript™ III reverse transcriptase (Thermo Fisher Scientific). The FastStart Universal SYBR Green Master (Roche, Basel, Switzerland) was used on the StepOnePlus™system (Life Technologies, Thermo Fisher Scientific) for quantitative PCR.(3; 4) Delta C_t values were calculated by subtracting the threshold cycle (C_t) value of the housekeeping gene (*ß-actin*) from the corresponding C_t value of the gene of interest. Relative transcript levels were determined with the $2^{-deltadeltaCt}$ method or expressed as $2^{-Ct(gene\text{-}Actb)}$.(5) To exclude that gene expression data were possibly distorted by variable *Actb* transcript levels in adipose tissue (6), we measured the C_t values of 18S ribsomal RNA (*Rn 18s*) in the epididymal WAT of wild-type and heterozygous *Wt1* knockout mice. For this purpose, first-strand cDNA synthesis was performed with random hexamer primers (Thermo Fisher Scientific) followed by RT-qPCR. Importantly, regression analysis revelaed a linear correlation (R^2=0.7926) between *Actb* mRNA and *Rn 18s* in murine epididymal WAT (ESM Fig. 1) indicating that *Actb* transcripts can be used for normalisation. Statistical significances were the same, no matter if *Actb* mRNA or *Rn 18s* were used for normalisation.

SDS-PAGE

Proteins obtained from different fat depots of wild-type and heterozygous *Wt1* knockout mice as well as from immortalised brown preadipocytes were blotted on PVDF membranes. The membranes were incubated overnight at 4°C with primary antibodies that were purchased from commercial suppliers and have been identified for the intended use. All antibodies were diluted in 2.5% nonfat milk (Carl Roth, Karlsruhe, Germany) in TBS/Tween: rabbit anti-WT1 (ab8990, Abcam, Cambridge, USA) diluted 1:1.000, rabbit anti-UCP1 (ab209483, Abcam) diluted 1:5.000, mouse anti-PPARγ (sc-7273, Santa Cruz Biotechnology, Dallas, Tx, USA) diluted 1:200. The primary antibodies were detected with HRP-coupled goat anti-rabbit IgG (sc-2004, Santa Cruz) and goat anti mouse IgG (Jackson 115-035-003), each one diluted 1:40.000, and visualized with WesternBright Sirius HRPsubstrate (Advansta, San José, CA, USA) following the manufacturer's description. The emitted chemiluminescence was detected with the ChemoCam imaging system (Intas Science Imaging, Göttingen, Germany). Equal protein loading was assessed with either mouse anti-GAPDH antibody (MAB374, diluted 1:2.000 Millipore, Burlington, MA, USA) or mouse anti-ACTIN antibody (MAB1501R, Millipore, diluted 1:10.000) after stripping of the membranes in 200 mmol/l NaOH for 5 min.(7)

Immunohistochemistry

Formalin-fixed, paraffin-embedded tissue sections (1.5 µm thick) from epididymal WAT and BAT of wild-type and heterozygous *Wt1* knockout mice were used for immunohistochemistry. Following deparaffinisation in Roticlear® solution (Carl Roth) and rehydration in ethanol (decreasing from 100% to 70%), antigen retrieval was performed with Target Retrieval Solution (Dako, Jena, Germany) in a steam cooker. The tissue sections were incubated overnight at 4°C with rabbit monoclonal anti-UCP1 antibody (ab209483, Abcam) diluted 1:4.000 in antibody diluent reagent solution (Cat. no. 00-2118, Invitrogen, Waltham, MA, USA). Immunoperoxidase activity was visualised using the Vectastain® Elite ABC kit (Vector Laboratories, Burlingame, CA, USA) according to the manufacturer's instructions. The slides were viewed under an inverse microscope (Eclipse Ti2, Nikon, Tokio, Japan) and photographed with the NIS-elements imaging software.(8) For the assessment of cell proliferation, immortalised brown preadipocytes (iBPC) with and without retroviral expression of WT1 were fixed in 4% formaldehyde and incubated overnight at 4°C with antibodies against the proliferation marker Ki-67 (SolA15, eBioscience, Santa Clara, CA, USA) and WT1 (ab 15249, Abcam), each used at a 1:100 dilution in antibody diluent reagent solution (Invitrogen). Goat anti-rat IgG F(ab)2'-fragment conjugated with Alexa Fluor 488 (112-226-003 Jackson ImmunoResearch Europe Ltd, Cambridgeshire, UK) and Cy3-conjugated donkey anti-rabbit IgG (711-165-152, Dianova, Hamburg, Germany) were used at a 1:200 dilution in antibody diluent reagent solution (Invitrogen) to label Ki-67 and WT1 expressing cells, respectively. Cell nuclei were counterstained with 4',6 Diamidino-2-phenyl-indol (DAPI, Sigma-Aldrich). More than 8.000 cells with and without expression of Ki-67 were counted in a blinded fashion using the trainable Weka segmentation tool within the Fiji ImageJ software.(9)

In addition, primary cells were isolated from the SVF of the epididymal WAT of either wild-type or heterozygous *Wt1* knockout mice (n=3, each). After 5 days in culture, the cells were seeded on coverslips and grown for another 24 hours. Formaldehyde fixation and immunolabelling for Ki-67 with DAPI counterstaining were performed as described for the iBPC. More than 8.000 primary cells were analysed.

Determination of the mean adipocyte area

Following deparaffinisation and rehydration, formalin-fixed, paraffin-embedded sections (4 µm thick) of epididymal fat were stained in Coomassie brilliant blue R-250 (Thermo Fisher Scientific) and scanned (10x lens) under an inverse microscope (Nikon, Eclipse Ti2) using the NIS-elements imaging software. Segmentation and analysis of the micrographs was performed

with Adiposoft and Fiji.(10) At least 2.500 adipocytes in 3 different tissue sections from each animal (n=10 in each group) were measured.

ESM References

1. Foryst-Ludwig A, Clemenz M, Hohmann S, Hartge M, Sprang C, Frost N, Krikov M, Bhanot S, Barros R, Morani A, Gustafsson JA, Unger T, Kintscher U: Metabolic actions of estrogen receptor beta (ERbeta) are mediated by a negative cross-talk with PPARgamma. PLoS Genet 2008;4:e1000108

2. Gohlke S, Zagoriy V, Cuadros Inostroza A, Meret M, Mancini C, Japtok L, Schumacher F, Kuhlow D, Graja A, Stephanowitz H, Jahnert M, Krause E, Wernitz A, Petzke KJ, Schurmann A, Kleuser B, Schulz TJ: Identification of functional lipid metabolism biomarkers of brown adipose tissue aging. Mol Metab 2019;24:1-17

3. Kirschner KM, Braun JF, Jacobi CL, Rudigier LJ, Persson AB, Scholz H: Amine oxidase copper-containing 1 (AOC1) is a downstream target gene of the Wilms tumor protein, WT1, during kidney development. J Biol Chem 2014;289:24452-24462

4. Martens LK, Kirschner KM, Warnecke C, Scholz H: Hypoxia-inducible factor-1 (HIF-1) is a transcriptional activator of the TrkB neurotrophin receptor gene. J Biol Chem 2007;282:14379-14388

5. Livak KJ, Schmittgen TD: Analysis of relative gene expression data using real-time quantitative PCR and the 2(-Delta Delta C(T)) Method. Methods 2001;25:402-408

6. Perez LJ, Rios L, Trivedi P, D'Souza K, Cowie A, Nzirorera C, Webster D, Brunt K, Legare JF, Hassan A, Kienesberger PC, Pulinilkunnil T: Validation of optimal reference genes for quantitative real time PCR in muscle and adipose tissue for obesity and diabetes research. Sci Rep 2017;7:3612

7. Muller M, Persson AB, Krueger K, Kirschner KM, Scholz H: The Wilms tumor protein WT1 stimulates transcription of the gene encoding insulin-like growth factor binding protein 5 (IGFBP5). Gene 2017;619:21-29

8. Kirschner KM, Sciesielski LK, Krueger K, Scholz H: Wilms tumor protein-dependent transcription of VEGF receptor 2 and hypoxia regulate expression of the testis-promoting gene Sox9 in murine embryonic gonads. J Biol Chem 2017;292:20281-20291

9. Arganda-Carreras I, Kaynig V, Rueden C, Eliceiri KW, Schindelin J, Cardona A, Sebastian Seung H: Trainable Weka Segmentation: a machine learning tool for microscopy pixel classification. Bioinformatics (Oxford, England) 2017;33:2424-2426

10. Galarraga M, Campión J, Muñoz-Barrutia A, Boqué N, Moreno H, Martínez JA, Milagro F, Ortiz-de-Solórzano C: Adiposoft: automated software for the analysis of white adipose tissue cellularity in histological sections. J Lipid Res 2012;53:2791-2796

Gene	ENSEMBL ID	Sequence (5' to 3')
Actb	ENSMUSG00000029580	Fw: CCGCGAGCACAGCTTCT
		Rev: GGGTACTTCAGGGTCAGGAT
Adrb3	ENSMUSG00000031489	Fw: GGCCCTCTCTAGTTCCCAG
		Rev: TAGCCATCAAACCTGTTGAGC
AdipoQ	ENSMUSG00000022878	Fw: TCCGGGACTCTACTACTTCTCTTACCAC
		Rev: GTCCCCATCCCCATACACCTG
Aldh1a1	ENSMUSG00000053279	Fw: AATCACCTCCATCTTGGTTGG
		Rev: CTGGGACACCCAGTGGA
Arbp1	ENSMUSG00000067274	Fw: TTTGGGCATCACCACGAAAA
		Rev: GGACACCCTCCAGAAAGCGA
Ccl2	ENSMUSG00000035385	Fw: AGGTGTCCCAAAGAAGCTGT
		Rev: AAGACCTTAGGGCAGATGCAG
Cebpa	ENSMUSG00000034957	Fw: ACATCAGCGCCTACATCGAC
		Rev: CTTGGCCTTCTCCTGCTGT
Cidea	ENSMUSG00000024526	Fw: AGGGACACCACGCATTTCAT
		Rev: CCGATTTCTTTGGTTGCTTG
Cpt1b	ENSMUSG00000078937	Fw: TACCGCATGGAGACATTGGC
		Rev: CCATGACCGGCTTGATCTCT
Ebf2	ENSMUSG00000022053	Fw: TGGCAGCATTGGAGAGTGAG
		Rev: AGAGCGCCAGGACAAAATGA
Fabp4	ENSMUSG00000062515	Fw: TGGAAGACAGCTCCTCCTCG
		Rev: AATCCCCATTTACGCTGATGATC
Fasn	ENSMUSG00000025153	Fw: ATCTCTCCAAGTTCGACGCC
		Rev: GGGCAATGCTTGGTCCTTTG
IL6	ENSMUSG00000025746	Fw: TAGTCCTTCCTACCCCAATTTCC
		Rev: TTGGTCCTTAGCCACTCCTTC
Leptin	ENSMUST00000169505.1	Fw: CAAGACCATTGTCACCAGGA
		Rev: TGAAGCCCAGGAATGAAGTC
Mrc1	ENSMUSG00000026712	Fw: GTCAGAACAGACTGCGTGGA
		Rev: AGGGATCGCCTGTTTTCCAG
Pck1	ENSMUSG00000027513	Fw: CAGCCGTCTGGCTAAGGAG
		Rev: CAGTAAACACCCCCATCGCTA
Pparg	ENSMUSG00000000440	Fw: TTGACAGGAAAGACAACGGA
		Rev: GAGCAGAGTCACTTGGTCATT
Ppargc1a	ENSMUSG00000029167	Fw: GAAAGGGCCAAACAGAGAGA
		Rev: GTAAATCACACGGCGCTCTT
Prdm16	ENSMUSG00000039410	Fw: TCTGCCACAAGTCCTACACG
		Rev: GAACATCTGCCCACAGTCCT
Rn18s	ENSMUSG00000119584	Fw: GATCAAAACCAACCCGGTCA
		Rev: CCGTTTCTCAGGCTCCCTCT
Saa3	ENSMUSG00000040026	Fw: TGACAGCCAAAGATGGGTCC
		Rev: ACTCATTGGCAAACTGGTCA
Slc2a4	ENSMUSG00000018566	Fw: CCCTCAAGCGGGTCTCACTA
		Rev: GTCACTCGCTGCCGAGG
Tmem26	ENSMUSG00000060044	Fw: ACCCTGTCATCCCACAGAG
		Rev: TGTTTGGTGGAGTCCTAAGGTC
Tnfrsf11b	ENSMUSG00000063727	Fw: AGTGTGAGGAAGGGCGTTAC
		Rev: AACTGTGTTTCGCTCTGGGG
Ucp1	ENSMUSG00000031710	Fw: CTGCCAGGACAGTACCCAAG
		Rev: CGTGGTCTCCCAGCATAGAA
Wt1	ENSMUSG00000016458	Fw: GATGTTCCCCAATGCGCCCTA
		Rev: TGCCCTTCTGTCCATTTCACT
Zfp423	ENSMUSG00000045333	Fw: CGCGATCGGTGAAAGTTGAAG
		Rev: TCCTTGCTGGAGGGAGATGA

Table 1: Primers used for qPCR

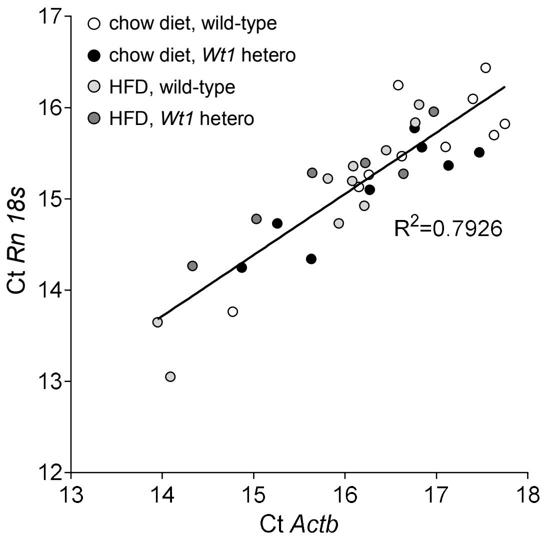

ESM Figure 1: Linear regression analysis of *Actb* mRNA and *Rn 18s* in epididymal WAT of mice.

Total RNA was isolated from epididymal WAT of wild-type and heterozygous *Wt1* knockout mice kept on either chow diet or high fat diet (HFD) for 11 weeks. Transcript levels were measured by RT-qPCR following first-strand cDNA synthesis.

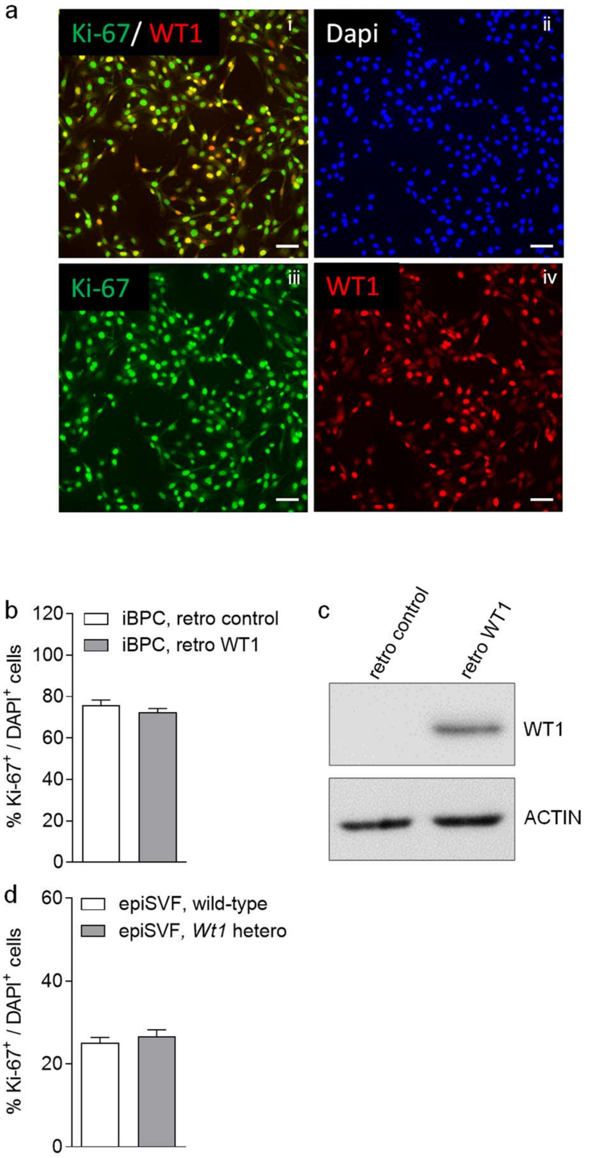

ESM Figure 2: Ki-67 labelling of preadipocytes

a. Representative Ki-67/WT1 double-immunofluorescent staining of immortalised brown preadipocytes (iBPC) with retroviral expression of WT1. Cells that co-express WT1 and Ki-67 can be distinguished by their yellow fluorescence in the merged image (i). Cell nuclei are visualized with DAPI (ii). b. Percentage of Ki-67-positive iBPC transduced with either WT1 expressing retrovirus or empty vector control. More than 8.000 cells totally were analysed. Bars indicate means±SEM (p>0.05, Student's t-test). c. Immunoblot of iBPC with (retro WT1) and without (retro control) retroviral expression of WT1. d. Percentage of Ki-67-positive SVF cells isolated and cultured from the epididymal WAT of either wild-type or heterozygous *Wt1* knockout mice (n=3, each). More than 8.000 cells totally were analysed. p>0.05, Student's t-test.

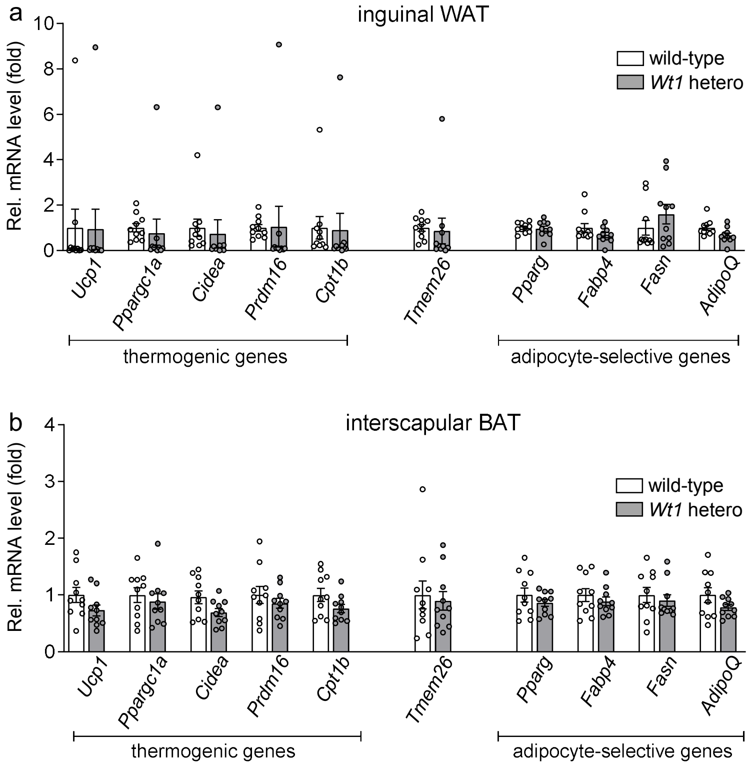

ESM Figure 3: Relative mRNA levels in inguinal WAT and interscapular BAT of wild-type and heterozygous *Wt1* knockout mice

Transcript levels in inguinal WAT (a) and interscapular BAT (b) were measured by RT-qPCR and normalised to *Actb* mRNA. Data are presented as fold difference *vs.* wild-type. Bars indicate means±SEM, n=10. No significant differences (p>0.05, Student's t-test) were detected between wild-type and heterozygous *Wt1* knockout mice (*Wt1* hetero). Note, that neither inguinal WAT nor interscapular BAT express *Wt1*.

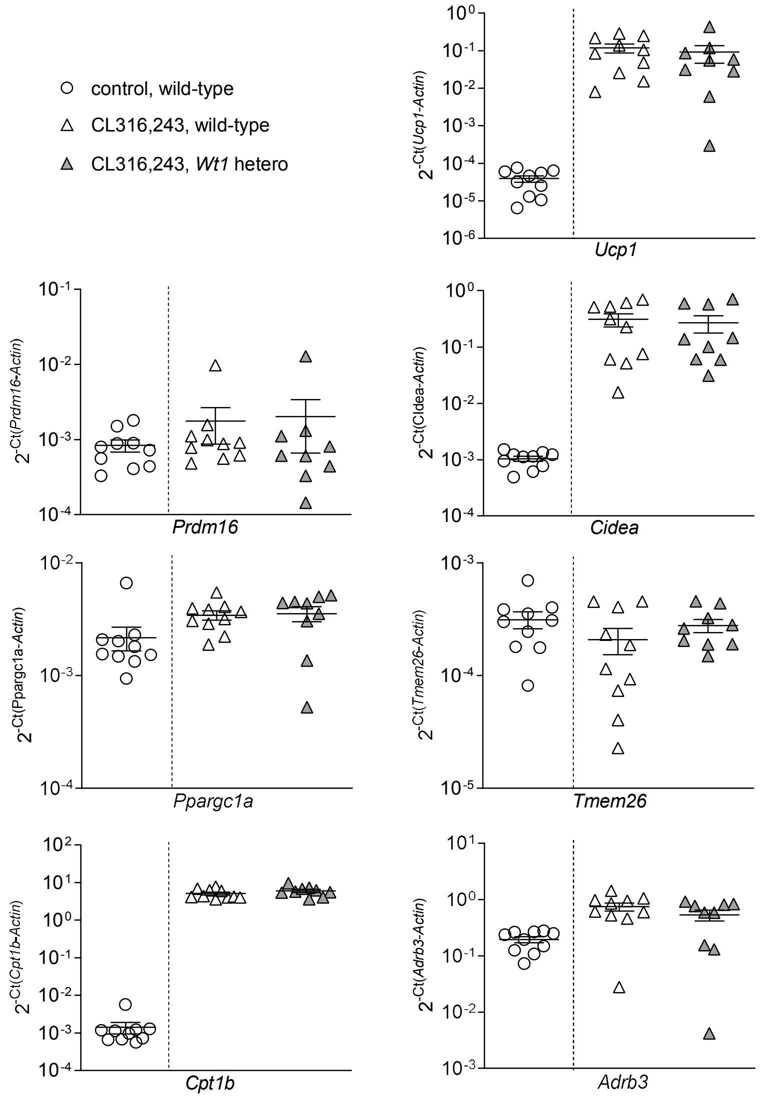

ESM Figure 4: Relative mRNA levels in epididymal WAT of wild-type and heterozygous *Wt1* knockout mice under β3-adrenergic stimulation

Wild-type and heterozygous *Wt1* knockout mice were injected intraperitoneally with the β3-adrenergic agonist CL316,243 (1 µg/ g body weight) on 10 consecutive days. Transcript levels in tissue specimens obtained from the epididymal WAT were measured by RT-qPCR. Values are depicted as $2^{-\text{deltaCt}}$, n=10 (wild-type mice), n=9 (*Wt1* heterozygous mice). No significant differences (p>0.05, Student's t-test) were detected between wild-type and heterozygous *Wt1* knockout mice (*Wt1* hetero) injected with CL316,243. Statistical differences between controls and mice receiving CL316,243 are not indicated.

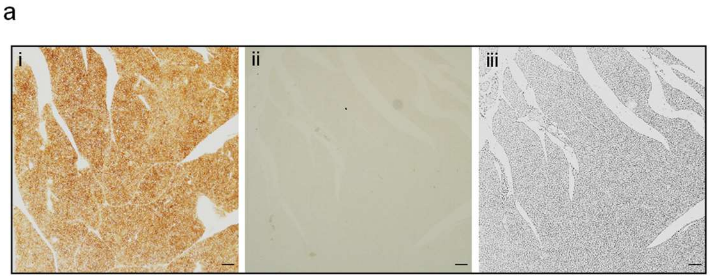

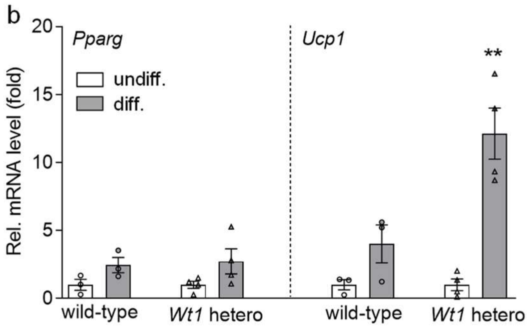

ESM Figure 5a: Representative UCP1 immunostaining in interscapular BAT of a wild-type mouse

Panel i: UCP1 antibody, panels ii and iii: serum control. Scale bars indicate 100 µm.

ESM Figure 5b: *Pparg* and *Ucp1* mRNA levels in SVF cells before and after treatment with differentiation cocktail

SVF cells were isolated from epididymal WAT of wild-type and heterozygous *Wt1* knockout mice and treated with differentiation cocktail for 7 days as described in ESM Methods. Transcripts were quantified by RT-qPCR and normalised to *Actb* mRNA. Data are presented as fold difference between differentiated (diff.) and undifferentiated (undiff.) cells in wild-type and *Wt1* mutant mice. Bars show means±SEM, n=3 (wild-type), n=4 (*Wt1* hetero). **p<0.01 between differentiated and undifferentiated cells, Student's t-test.

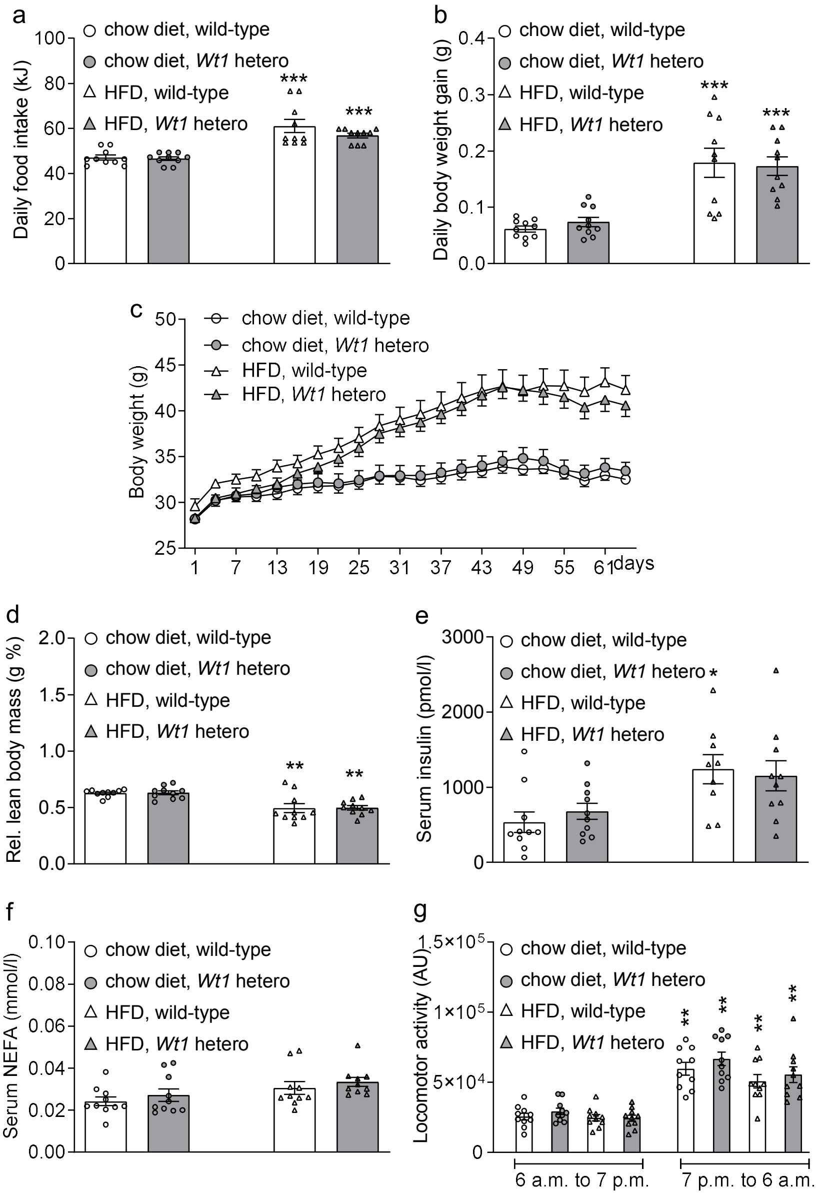

ESM Figure 6: Effects of HFD feeding in wild-type and heterozygous *Wt1* knockout mice Wild-type and heterozygous *Wt1* knockout mice (n=40 total) were kept on either chow diet (10% of kJ from fat), or high fat diet (HFD, 60% of kJ from fat) for 11 weeks. Daily food intake (a), daily body weight gain (b), body weight during the entire feeding period (c), relative lean body mass (d), serum insulin concentration (e) and serum concentration of non-esterified fatty acids (NEFA) (f) are shown. Locomotor activity of wild-type and *Wt1* heterozygous mice is depicted in arbitrary units (AU) (g). Bars indicate means±SEM, n=10 in each group. **p*<0.05, ***p*<0.01, ****p*<0.0001 between mice on HFD and chow diet, ANOVA with Tukey post-hoc test. Note, that no statistical differences between wild-type and heterozygous *Wt1* knockout mice were observed.

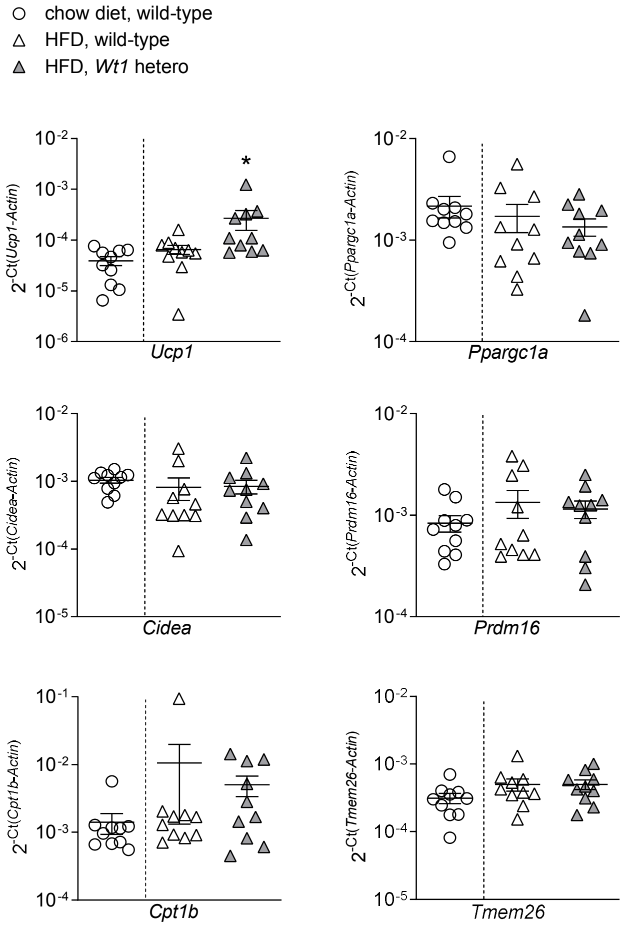

ESM Figure 7: Relative transcript levels of thermogenic genes in epididymal WAT of wild-type and heterozygous *Wt1* knockout mice receiving HFD.

Wild-type and heterozygous *Wt1* knockout mice (n=40 total) were kept on HFD (60% of kJ from fat, 21.92 kJ/g chow) for 11 weeks. Transcript levels in epididymal WAT were measured by RT-qPCR and normalised to *Actb* mRNA. Data are presented as $2^{-\text{deltaCt}}$, n=10 each. *p<0.05, between wild-type and heterozygous *Wt1* knockout mice receiving HFD, Student's *t*-test.
